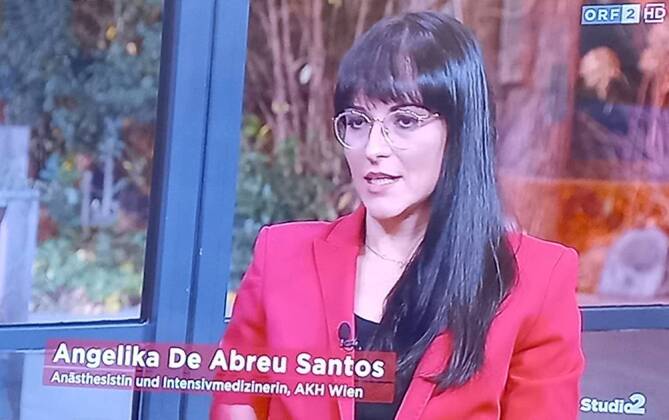# Videoappelle der ARGE Junge Anästhesie – ein Resümee

**DOI:** 10.1007/s44179-022-00011-3

**Published:** 2022-02-14

**Authors:** Rebana Scherzer

**Affiliations:** 1 Universitätsklinik für Anästhesie, Allgemeine Intensivmedizin und Schmerztherapie, MedUni Wien/AKH Wien, Wien, Österreich; 2 ARGE Junge Anästhesie, ÖGARI, Wien, Österreich

Die Impfappelle der ARGE Junge Anästhesie führten zu einem hohen öffentlichen Interesse und wurden in den sozialen Medien tausendfach geteilt und aufgerufen. Wie es dazu kam und aus welchen Beweggründen heraus die Aktion ins Leben gerufen wurde, erläutert die Initiatorin der Aktion.

In Zeiten, in denen die Entscheidung für oder wider die Impfung nicht mehr aus gesundheitlichen Gründen, sondern oftmals aus politischen oder anderen Überlegungen getroffen wird, ergab sich die Frage, auf welche Art und Weise der Bevölkerung die Impfdringlichkeit nahegebracht werden könnte. Zusätzlich zeigte sich über die letzten Monate hinweg ein weiteres Problem, nämlich die niedrige Durchimpfungsrate in den jüngeren Jahrgängen und die sich zunehmend häufenden schweren Verläufe in dieser Altersklasse. Jung, gesund und ohne Vorerkrankungen boten keine Garantie mehr dafür, nicht schwer am SARS-CoV-2-Virus zu erkranken.

Dies spiegelte sich auch auf unseren Intensivstationen wider. Waren es in den ersten Wellen vorwiegend ältere Patient*innen, für die eine intensivmedizinische Betreuung nötig war, so zeichnete sich in den darauffolgenden Wellen auf den Intensivstationen ein gänzlich anderes Bild ab.

Daraus entstand die Idee, gerade die jüngere Bevölkerung in Form von Videoappellen von jungen Assistenzärzt*innen und jungen Fachärzt*innen, die auf COVID-Intensivstationen arbeiten, zu erreichen und direkt anzusprechen. Fundierte Aufklärungsversuche auf Augenhöhe – von Jung zu Jung – folgten, getätigt von jenen, die tagtäglich an vorderster Front gegen das Virus kämpfen, das weder Alter noch politische Gesinnung kennt.

## Junge Held*innen vor den Vorhang!

Den jungen Kolleg*innen wurde eine Plattform geboten, um über ihre mitunter sehr tragischen Erfahrungen berichten zu können. Die Betreuung schwerstkranker Patient*innen ist nie eine leichte, doch gerade in der Ausbildungszeit zur Fachärztin und zum Facharzt für Anästhesie und Intensivmedizin, in der Erfahrungsschätze erst gesammelt werden, kann die tagtägliche Arbeit mit Schwerkranken besonders zehren, insbesondere während einer Viruspandemie, die nunmehr schon fast zwei Jahre andauert. Dem hinzuzufügen ist das steigende Unverständnis und die steigende Frustration über ungeimpfte Patient*innen.

In den ersten Wellen beziehungsweise vor der Möglichkeit einer Impfung waren schwere Verläufe schicksalshaft, doch nun stehen Kolleg*innen und unsere hochgeschätzte Intensivpflege schwer Erkrankten und Sterbenden gegenüber, deren Verlauf mit der Impfung deutlich hätte abgemildert werden können und eine intensivmedizinische Betreuung in gut 90 % der Fälle nie notwendig geworden wäre.

Nichtsdestotrotz werden alle Patient*innen mit höchster Professionalität gleich behandelt.

## In den Medien

Umso erfreulicher war es, dass sich die Aktion wie ein Lauffeuer in den öffentlichen und sozialen Medien verbreitete. Auftritte in der „ZIB Nacht“ im ORF (Dr. Paul Köglberger), im „Studio 2“ (Dr. Angelika De Abreu Santos), bei „Wien Heute“ (Dr. Jakob Vogel), „Talk 1“ (DGKP Elias Jandl) und Artikel in den Printmedien „Die Presse am Sonntag“ (Interviews mit Dr. Margarete Steiner, Dr. Paul Köglberger und DGKP Stefan Kahri), „Der Standard“ und „Heute“ – um nur einige zu nennen – waren die positive Folge.

Somit ist es uns gelungen, öffentliche Aufmerksamkeit für diese Problematik zu wecken. Die Wahrheit ist dem Menschen zumutbar (Ingeborg Bachmann), und möglicherweise gelingt es nur so, die bislang noch skeptische Bevölkerung dazu zu motivieren, die Impfangebote anzunehmen und somit aktiv und solidarisch die Bekämpfung der Pandemie mitzutragen.

## Ein Dankeschön

Wurden wir zu Beginn der Pandemie als Gesundheitspersonal beklatscht, erscheint es nun so, als ob die Sympathien der Bevölkerung uns gegenüber nachlassen. Es steht eine kleine, laute Minderheit, die vor unseren Spitälern demonstriert, uns auspfeift und beschimpft, einer doch viel größeren schweigenden Mehrheit der Bevölkerung gegenüber.

Das kratzt an unseren Nerven und nagt an unserer Motivation. Daher möchten wir, die ARGE Junge Anästhesie, diese Gelegenheit auch dafür nutzen, um all jenen DANKE zu sagen, die seit Beginn der Pandemie unermüdlich und mit enormer Aufopferungsbereitschaft für unsere Patient*innen kämpfen und nahezu Menschenunmögliches leisten, damit die Versorgung all unserer Patient*innen bestmöglich aufrechterhalten werden kann! Besondere Anerkennung verdienen unsere jungen Kolleg*innen, die noch am Anfang ihres medizinischen Werdegangs stehen. Sie werden bei der Pandemiebekämpfung sowohl menschlich als auch fachlich massiv gefordert und meistern dies bravourös.

Auch wenn es manchmal so scheint, als wäre kein Ende in Sicht, die Pandemie wird vorübergehen – halten wir zusammen und halten wir durch!

**Figure 1:**